# Sensory brain activation during rectal balloon distention: a pilot study in healthy volunteers to assess safety and feasibility at 1.5T

**DOI:** 10.1007/s10334-022-01044-0

**Published:** 2022-10-13

**Authors:** Roman Assmann, Sanne Rutten, Job van den Hurk, Sadé Laurèl Assmann, Paul Janssen, Nicole Bouvy, Jarno Melenhorst, Stephanie Breukink

**Affiliations:** 1 Department of Surgery, Maastricht University Medical Center, Maastricht, The Netherlands; 2 Department of Translational Neuroscience, School for Mental Health and Neuroscience (MHeNS), Maastricht University, Maastricht, The Netherlands; 3 Scannexus, Maastricht, The Netherlands; 4 NUTRIM, School of Nutrition and Translational Research in Metabolism, Maastricht, The Netherlands; 5 GROW, School for Oncology and Developmental Biology, Maastricht, The Netherlands

**Keywords:** Magnetic resonance imaging, Fecal incontinence, Rectum

## Abstract

**Objective:**

Although increasing evidence suggests a central mechanism of action for sacral neuromodulation, the exact mechanism remains unclear. We set up a scanning paradigm to measure brain activation related to various stages of rectal filling using rectal balloon distention.

**Materials and Methods:**

Six healthy volunteers underwent rectal balloon distention during MRI scanning at a 1.5T scanner with a Tx/Rx head coil. MR images were collected at four levels of distention: empty balloon (EB), first sensation volume (FSV), desire to defecate volume (DDV), maximum tolerable volume (MTV). Data were analyzed using BrainVoyager 20.4. Whole brain and ROI-based fixed-effects general linear model analyses were performed on the fMRI time-course data from all participants.

**Results:**

Rectal filling until FSV evoked the most blood-oxygen-level-dependent responses in several clusters throughout the cortex, followed by the responses evoked by rectal filling until DDV. Interestingly, rectal filling until MTV evoked negative responses compared to baseline throughout the cortex. No negative side effects were found.

**Discussion:**

This study shows that a standardized paradigm for functional MRI combined with rectal filling is feasible and safe in healthy volunteers and is ready to be used in fecal incontinent patients to assess whether their brain activity differs from healthy controls.

## Introduction

In healthy individuals, the defecation process starts when luminal contents are propelled forwards by colonic activity, filling the rectum [1]. Rectal filling induces the rectoanal inhibitory reflex (RAIR), which relaxes the internal anal sphincter (IAS). Upon relaxation of the IAS, distal rectal contents are moved to the upper anal canal, where the nature of rectal contents can be differentiated [2]. In case defecation is convenient, the pelvic floor and external anal sphincter (EAS) are relaxed and defecation occurs. In case defecation is not convenient, the pelvic floor muscles and external sphincter are contracted, thereby deferring defecation. The ability to sufficiently contract the pelvic floor muscles, combined with adequate anorectal sensation are the cornerstones of being continent [3]. In fecal incontinence (FI) patients, one of these mechanisms, or a combination of both, is absent, but the underlying relationship remains complex and multifactorial.

Throughout the past two decades, sacral neuromodulation (SNM) has demonstrated to be an effective treatment option for intractable fecal incontinence [4–10]. Although there is increasing evidence for a central mechanism of action of SNM on FI, the exact mechanism responsible for the beneficial effect remains unclear [11–14]. To clarify the possible central working mechanisms of SNM in patients with FI, the exact central neural mechanisms involved in the sensation of rectal filling need to be identified.

Functional magnetic resonance imaging (fMRI) is a method that is sensitive to fluctuations in blood oxygen levels in the brain’s vascular system. These changes in blood oxygen levels are closely related to synaptic input activity [15, 16].

To date, it is unknown how neural responses associated with rectal sensation differ between healthy controls and FI patients. Therefore, setting up experimental designs similar to studies performed on urinary incontinence (UI) and to studies in which sensation or pain is exerted through rectal balloon distention may be fruitful [11, 17]. In patients with UI, bladder fullness enhances activity in several cortical regions, most prominently in the midbrain and limbic cortical areas [17]. In studies in which sensation or pain was exerted through rectal balloon distention, changes in evoked neural responses were found in several areas including the pre- and postcentral gyrus, thalamus, primary somatosensory (SI), secondary somatosensory cortex (SII), sensory association cortex, anterior cingulate cortex (ACC) and the insular cortex [18–20]. These studies showed that specific brain regions are related to perceptions of bladder fullness and rectal sensation/pain. We assume that comparable neural mechanisms are involved in the perception of rectal filling. To study these mechanisms, a standardized scanning paradigm to evaluate evoked neural responses during rectal filling stages should be available. We, therefore, designed such a paradigm to measure neural responses during the following rectal filling stages: first sensation volume (FSV), desire to defecate volume (DDV) and maximum tolerable volume (MTV) of the rectum.

The aim of this feasibility and safety pilot study was to evaluate whether this scanning paradigm was successful at assessing evoked neural responses related to various stages of rectal filling in healthy volunteers. All volunteers underwent a 1.5T MRI with a Tx/Rx head coil. Moreover, the pain scores of participants were determined on a visual analog scale (VAS) to establish the safety of the scanning paradigm.

We studied brain activation levels in the following bilateral regions-of-interest (ROIs): anterior cingulate gyrus, insular cortex, precentral gyrus, postcentral gyrus, thalamus and the pons. We hypothesized that different rectal filling stages would evoke different activation levels within these areas.

## Materials and methods

### Participants and ethical statement

Six healthy volunteers were recruited through public advertising (1 female, mean age 42.5 years, range: 20–70 years). All participants were fecal continent and without a history of inflammatory bowel disease, neurological, psychiatric, kidney or cardiac disorders. The approval for the study was granted by the Ethical Committee of Maastricht University Medical Center (MUMC + , Maastricht, the Netherlands). Clinical Trial Center Maastricht independently monitored this study. All participants gave written informed consent to participate and to publish, prior to participating in the study.

### Experimental design

**Fig. 1:**
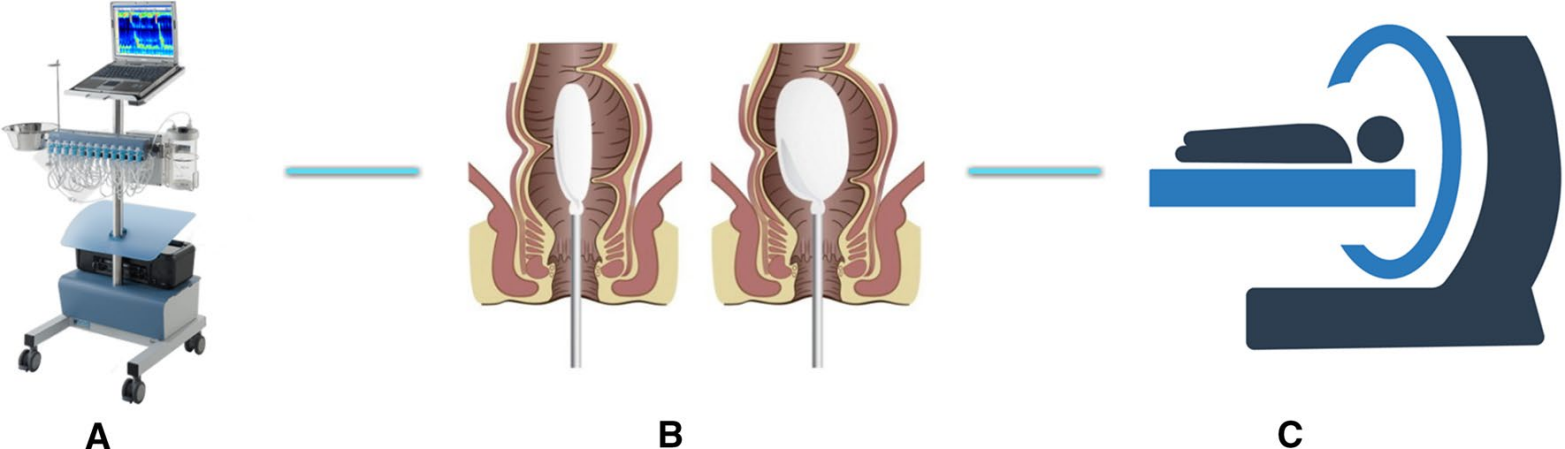
Schematic depiction of the experimental setup

Prior to the start of the MRI scanning, a rectal balloon was inflated, using the Solar GI, to determine the following four levels of distention thresholds in each participant as per the International Anorectal Physiology Working Group (IAPWG) recommendations [21]: empty balloon (EB), first sensation volume (FSV), desire to defecate volume (DDV) and maximum tolerable volume (MTV). These volumes were determined while the participants were in the MRI scanner shortly before the start of the experiment. After positioning the rectal balloon, subjects were positioned in the supine position, with their knees bent on a triangular pillow and covered with a blanket. Their heads were placed in a head coil. A schematic depiction of the experimental setup with the Solar GI (Fig. 1A), the rectal balloon (Fig. 1B) and the MRI (Fig. 1C) can be found in Fig. 1.

**Fig. 2:**
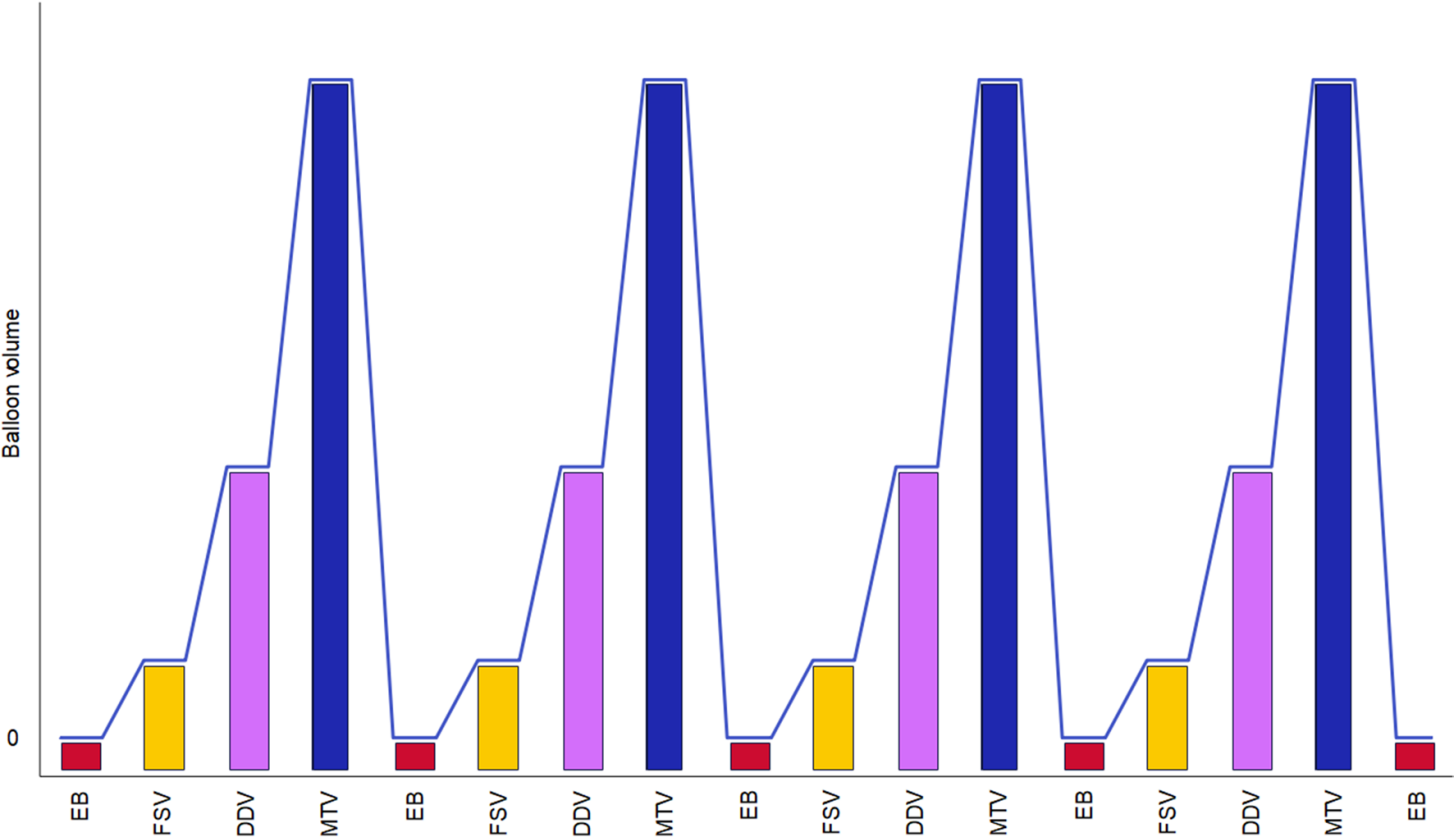
Schematic depiction of rectal filling stages

The experiment consisted of 4 functional runs that lasted about 10 min each (Fig. 2). Blood-oxygen-level-dependent (BOLD) responses were measured during the following staircase cycle: EB, inflation level 1, FSV, inflation level 2, DDV, inflation level 3, MTV and deflation. This cycle was repeated 4 times within one. Each run ended with an extra EB measurement. Each EB measurement lasted 20 s, each FSV, DDV or MTV measurement lasted 12 s and each deflation measurement lasted 40 s. The filling periods varied from 1 to 7 s, during these periods the balloon was gradually inflated until it reached the participants’ individual filling threshold for the corresponding condition. A vacuum pump was used for the deflation of the balloon. Ultimately, we collected 16 repetitions (12 s for each repetition, which corresponds to 4 volumes) for each of the filling conditions (FSV, DDV and MTV).

Prior to the fMRI measurements, anatomical images were collected for each participant. The entire experiment was performed within 45 min, which was easily feasible for all participants. For one of the participants, three runs were collected instead of four, due to technical failure.

Given the expected low neural responses to the subtle stimulation, we aimed to collect as many repetitions as possible in a 1 hour scanning period. This entails that we chose to fill up the balloon in an increasing stepwise fashion (staircase approach), rather than pseudo-randomizing the trials. Pseudo-randomizing would take more time since it would require deflating the balloon between each condition. Not only would this be unfavorable for scanning time, but this would also be detrimental to the participants’ comfort.

### Safety

Within 5 min after concluding the experiment, participants were asked to rate their maximum, minimum and current pain score during the experiment on a 10-point VAS scale. Additionally, participants were called 48 h after the experiment to determine whether any discomfort had occurred after leaving the hospital.

### MRI parameters

Brain imaging was performed on a 1.5T scanner (Ingenia; Philips Medical Systems, Best, the Netherlands) with a transmit and receive head coil (Philips dStream T/R head coil) at the Maastricht University Medical Center (MUMC + , Maastricht, the Netherlands). The functional T2*-weighted images were acquired using a multi-shot Echo Planar Imaging (EPI) sequence (repetition time/time of acquisition [TR] = 3000 ms; echo time [TE] = 50 ms, voxel size = 1.8 × 1.8 × 4 mm). Each volume consisted of 31 slices covering the whole brain. Anatomical T1-weighted images were acquired using the 3D-NAFTRA sequence (voxel size = 1.0 × 1.0 × 1.0 mm).

### Data preprocessing

Functional and anatomical images were analyzed using BrainVoyager 20.4 [24]. Preprocessing steps for the functional images consisted of slice scan-time correction (cubic spline interpolation), 3D-motion correction (trilinear interpolation for motion estimation/sinc interpolation for correction) and temporal high-pass filtering to remove low-frequency drifts (of maximum four cycles per time course/run). The functional images were co-registered to the anatomical images, and both were transformed into MNI space.

### Anatomical ROIs

**Fig. 3:**
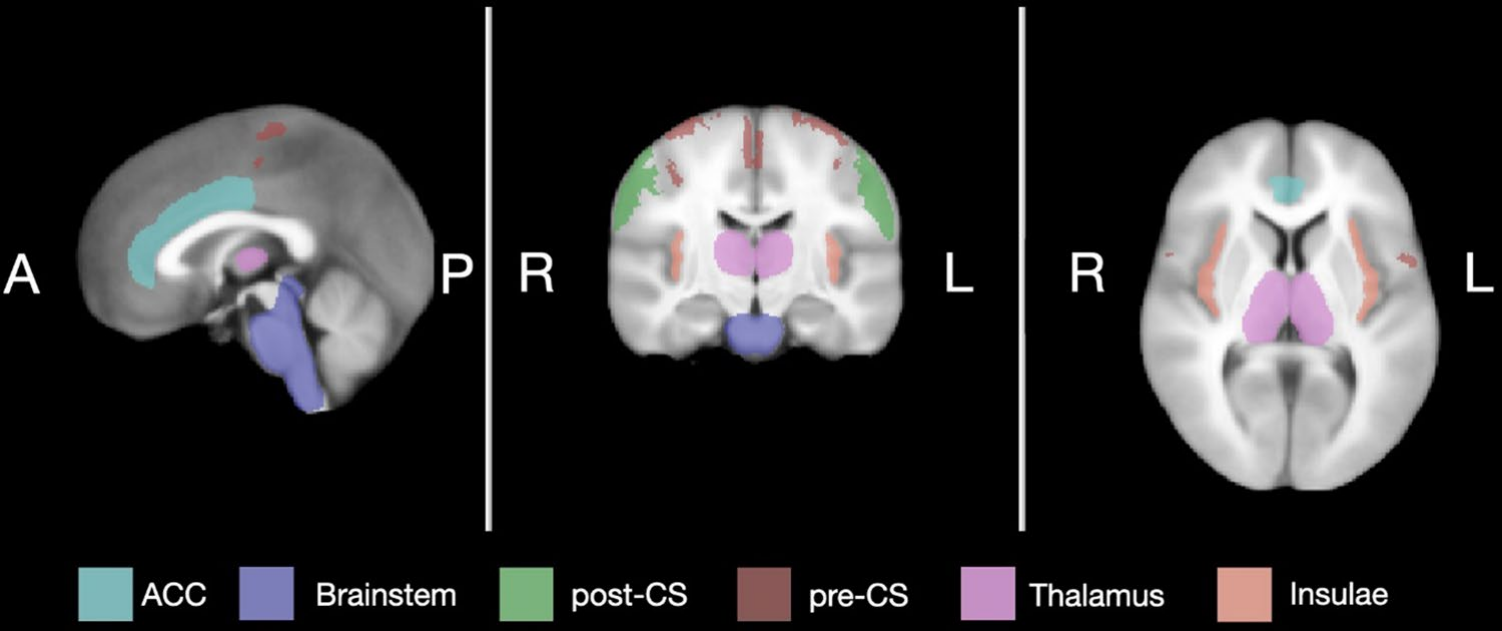
Regions of interest

Region-based group analyses were performed to assess the differences in activation levels between the three filling conditions. The following regions of interest (ROIs) were obtained from Harvard- Oxford Cortical Structural and Harvard–Oxford Subcortical Structural atlases as implemented in Functional Magnetic Resonance Imaging of the Brain (FMRIB) Software Library (FSL) [25]: anterior cingulate gyrus, insular cortex, precentral gyrus, postcentral gyrus, thalamus and brainstem. To examine responses within a subregion of the brainstem, the pons was manually segmented from this structure using BrainVoyager and added as an additional ROI to the analyses. Since the pontine micturition center can be found in the pons (include reference), we hypothesized a similar structure might be present to control defecation. All ROIs were segmented in left- and right hemispheric portions by following the midsagittal plane along the MNI brain atlas (Fig. 3).

### fMRI statistical analysis

Whole brain and ROI-based fixed-effects general linear model (GLM) analyses were performed on the fMRI time courses from all the participants. We used one predictor per condition (EB, FSV, DDV and MTV; convolved with a double gamma hemodynamic response function). Inflation and deflation measurements were included as confounding predictors.

Whole brain contrast maps (*t* statistics) were calculated to estimate evoked-neural responses to the separate rectal filling conditions throughout the entire brain. All contrast maps were thresholded with uncorrected  = *p* value < 0.05 and a cluster-size threshold of 10 voxels. Per ROI, contrast maps were calculated to assess the differences in evoked responses between the different conditions. This was done for each hemisphere separately. For each ROI we tested the following contrasts: c_1_: FSV > DDV, c_2_: FSV > MTV, c_3_: DDV > MTV. The contrasts were corrected for multiple comparisons using false discovery rate (FDR)-correction ( = *p *FDR < 0.05; [26], as implemented in Matlab (www.mathworks.com)).

## Results

None of the volunteers reported any pain or negative side effects related to the experiment.

### Cortical responses during different rectal filling stages

**Fig. 4:**
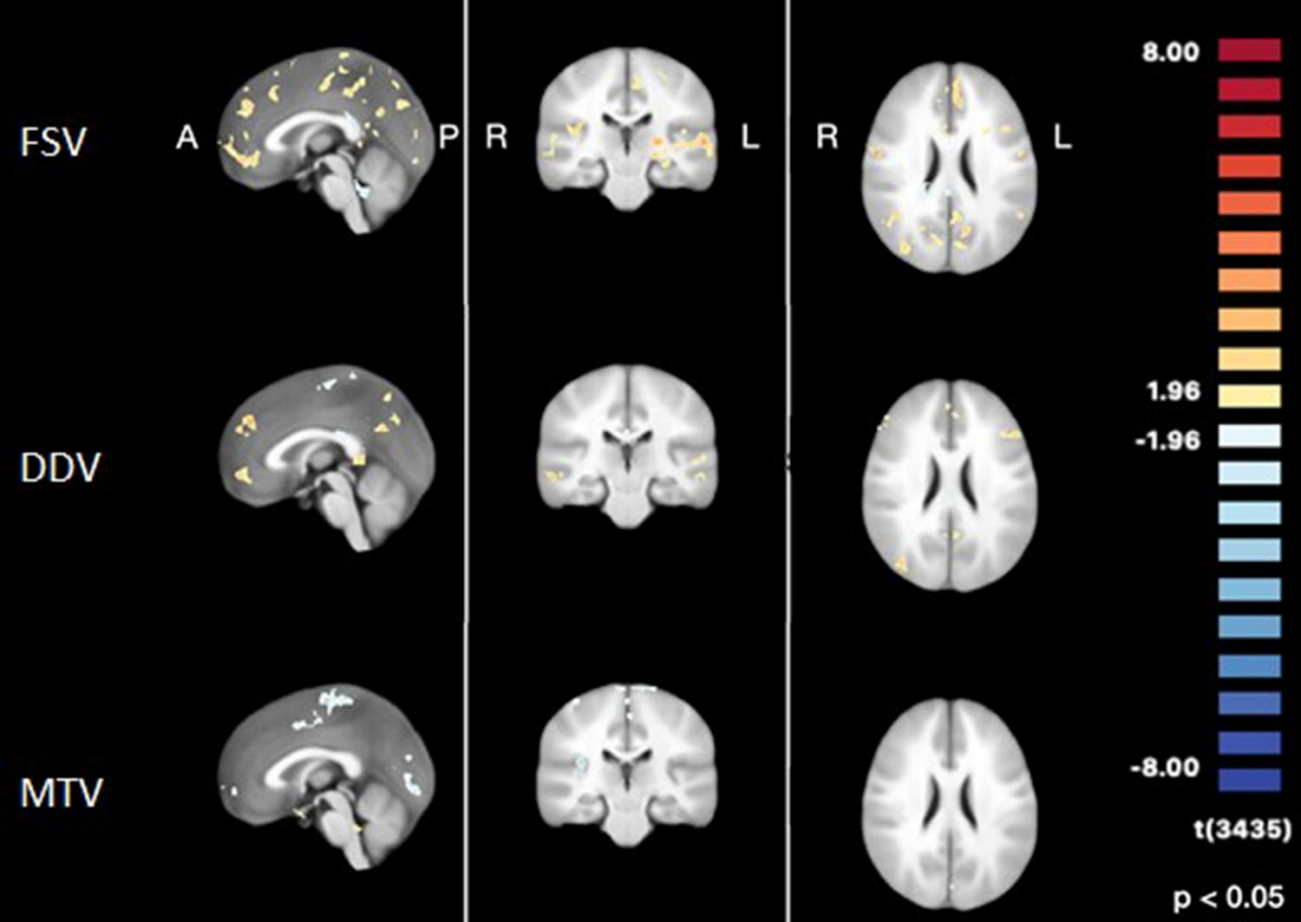
Evoked neural responses during different rectal filling stages

We measured the evoked-neural responses during different rectal filling stages. Generally, rectal filling until FSV evoked most uncorrected BOLD responses in several clusters throughout the cortex, followed by the responses evoked by rectal filling until a desire to defecate. Interestingly, rectal filling until MTV evoked negative responses compared to baseline (empty balloon) throughout the cortex (uncorrected *p* value < 0.05 with clustersize-thresholding of 10 voxels; Fig. 4).

### Differences in ROI-based cortical responses between rectal filling stages

Within the ROIs, we did not find differences in evoked responses between FSV and DDV (c_1_: FSV > DDV). We did find significant differences between FSV and MTV (c_2_: FSV > MTV) within the left precentral gyrus (*t*_*(*95)_ = 4.5, pFDR = 0.001) and left postcentral gyrus (*t*_*(*95)_ = 5.5, pFDR < 0.001). Additionally, we found differences between DDV and MTV (c_3_: DDV > MTV) in the following ROIs: bilateral insular cortex (left: *t*_*(*95)_ = 2.7, pFDR = 0.010; right: *t*_*(*95)_ = 4.0, pFDR = 0.009), precentral gyri (left: *t*_*(*95)_ = 4.5, pFDR < 0.001; right: *t*_*(*95)_ = 4.4, pFDR < 0.001), postcentral gyri (left: *t*_*(*95)_ = 5.5, pFDR < 0.001; right: *t*_*(*95)_ = 3.9, pFDR < 0.001), pons (left: *t*_*(*95)_ = -3.3, pFDR = 0.002; right: *t*_*(*95)_ = -2.8, pFDR = 0.008), right anterior cingulate cortex (*t*_*(*95)_ = 2.6, pFDR = 0.010) and left thalamus (*t*_*(*95)_ = 2.2, pFDR = 0.030).

### Safety

Average current pain scores, which were obtained 5 min after concluding the experiment, was 0.38 (range 0–1) on a 10-point VAS scale. During the experiment, the maximum pain score was 3.77 (range 0–7) and the minimum pain score was 0.87 (range 0–3). Participants with a higher than average pain score all mentioned that the sudden distention of the balloon surprised them, causing discomfort, even after explicit explanation before the study that this could occur. No adverse events occurred during the study. Moreover, no experiences of pain or discomfort came to light during the telephone conversations 48 h after the experiment.

## Discussion

In this pilot study, we evaluated the feasibility and safety of a standardized scanning paradigm to measure evoked neural responses during rectal filling in healthy volunteers. In addition to the feasibility, we explored which brain regions elicited different neural responses during rectal filling.

Most uncorrected BOLD responses were found in several clusters throughout the cortex when filling the rectum until FSV when compared to baseline. Rectal filling until DDV evoked less BOLD responses than filling until FSV, although this decrease did not reach statistical significance. Filling until MTV evoked even less BOLD responses than filling until DDV in several regions. Thus, it seems like most brain regions are being activated during the first stage of rectal filling. BOLD responses even showed a deactivation of several cortical and subcortical regions, such as the left pre- and postcentral gyri, bilateral insular cortex, pre- and postcentral gyri and pons, right anterior cingulate cortex and left thalamus, during maximum filling of the rectum. Although the BOLD response shows a downward trend with increasing rectal pressure, the magnitude of the negative peak in the MTV condition is larger than expected, i.e., lower than baseline, by the increase in rectal pressure. This implies that other explanations, both physiological as well as methodological, are to be sought for this effect. On the physiological level, one could argue that the maximum tolerable pressure differs from the other conditions in that it induces pain. Kong and collegues showed that pain can lead to deactivation in several brain areas involved in the so-called pain matrix, which comprises regions such as the thalamus, insula, and postcentral gyri [27]. Therefore, it is not unlikely that painful sensations induced by the maximum filling of the rectum directly involved regions in the pain matrix. A methodological explanation for this negative peak in the MTV condition would be that this condition always followed FSV and DDV, without deflating the balloon. Therefore, a possible effect of these preceding conditions on MTV cannot be ruled out. The staircase setup of the scanning paradigm was used for time reasons since it allowed for more scanning time per condition, and to minimize participant discomfort. However, since the order of conditions was identical for each cycle, each filling state was always preceded by the same filling state. Moreover, for time reasons, no baseline measurement was conducted in between each condition. Baseline brain activity was only measured before and after each staircase cycle. Although this is not an uncommon procedure when one is interested in the relative difference between conditions, a randomization of the trial order would have allowed for better response estimates.

The low pain scores, and absence of adverse events or discomfort after concluding the experiment, showed that this scanning paradigm is safe to use. In the case of a follow-up study, it would be advisable to explain very explicitly to participants that the sudden distention of the balloon might initially surprise them.

The lead (model 3889/3093) and implantable pulse generator (IPG, model 3058) produced by Medtronic, which were used in the majority of SNM patients up until 2020, received FDA conditional MRI approval at 1.5T with a transmit/receive (Tx/Rx) head coil [22, 23]. Therefore, to allow for follow-up studies with patients having an IPG in place, we deliberately chose to use a scanner with this field strength. However, this inherently yields several limitations. First, 1.5T MRI has limited spatial resolution per time unit compared to higher magnetic field strengths. Second, the Tx/Rx coils that are approved for use on patients with aforementioned implants are typically restricted in transmit power, limiting signal-to-noise ratio. Third, one could hypothesize that nuclei in the brain stem are of interest in this line of research, given this brain region’s involvement in for instance micturition. At 1.5 T, with limited transmit capabilities, these deep-lying brain regions are likely too small for the relatively large voxel size, as well as too far away from the surface to yield a reliable signal. Lastly, the long repetition time (TR) to acquire a particular spatial resolution led to a long scanning time per volume. Since total scanning time was limited, this resulted in the staircase approach of the scanning paradigm previously mentioned, whereas a randomized stimulation protocol would be preferable. These factors need to be taken into consideration when designing an experiment on this specific participant population.

In this study, we wanted to evaluate whether we could differentiate brain activity between EB, FSV, DDV and MTV. Therefore, we needed a controlled situation and due to technical limitations of our MRI, we placed our participants in a supine position. We realize that with this study setup, outcomes may not perfectly reflect body position throughout the day in real life. We know that gravity in daily life has an effect on the filling status of the rectum. This implies that in theory, the DDV in the supine position is larger than in the upright position. For example, in runners, it is very common to have bowel problems during running, but when they stop running and sit down, these problems disappear. Additionally, most people do not have problems holding their stool during the night.

Upon completion of this study, Medtronic introduced new SNM leads and IPGs, which are suitable for 3 T MRI. Therefore, future studies using 3 T MRI can benefit from a shorter repetition time (TR) to acquire a particular resolution, leading to a decreased time needed for scanning. Shorter repetition time might result in higher T1- weighted images, however, this will not compromise the ability to detect BOLD fluctuations which depends on T2^(*)^ fluctuations. Consequently, the possibility to randomize conditions without sacrificing statistical power becomes feasible.

Given the relatively lower SNR that can be achieved at 1.5 T compared to higher magnetic field strengths, compromises in terms of slice thickness had to be made to favour a higher in-plane resolution. However, future studies at higher field strengths can benefit from the increase in SNR to acquire isotropic voxels at a high resolution, to improve spatial specificity in all 3 dimensions and to reduce partial voluming effects caused by increased slice thickness.

In conclusion, this study showed that a standardized scanning paradigm for functional MR imaging combined with rectal filling is feasible and safe. New developments within SNM MR compatibility make this an interesting prospect for future studies. The next step would be to use an optimized (non-staircase) scanning paradigm based on the one discussed in this paper and apply it to fecal incontinent patients to assess if their brain activity differs from healthy controls and if so, to establish the nature of these differences.
